# Combined approach for gynecomastia

**DOI:** 10.3205/iprs000089

**Published:** 2016-02-23

**Authors:** Ahmed Hassan El-Sabbagh

**Affiliations:** 1 Plastic Surgery Center, Faculty of Medicine, Mansoura University, Mansoura, Egypt

**Keywords:** gynecomastia, liposuction, periareolar incision, surgical excision

## Abstract

**Background:** Gynecomastia is a deformity of male chest. Treatment of gynecomastia varied from direct surgical excision to other techniques (mainly liposuction) to a combination of both. Skin excision is done according to the grade. In this study, experience of using liposuction adjuvant to surgical excision was described.

**Patients and methods:** Between September 2012 and April 2015, a total of 14 patients were treated with liposuction and surgical excision through a periareolar incision. Preoperative evaluation was done in all cases to exclude any underlying cause of gynecomastia.

**Results:** All fourteen patients were treated bilaterally (28 breast tissues). Their ages ranged between 13 and 33 years. Two patients were classified as grade I, and four as grade IIa, IIb or III, respectively. The first 3 patients showed seroma. Partial superficial epidermolysis of areola occurred in 2 cases. Superficial infection of incision occurred in one case and was treated conservatively.

**Conclusion:** All grades of gynecomastia were managed by the same approach. Skin excision was added to a patient that had severe skin excess with limited activity and bad skin complexion. No cases required another setting or asked for 2^nd^ opinion.

## Zusammenfassung

**Hintergrund:** Gynäkomastie ist eine Deformität der männlichen Brust. Die Behandlung reicht von der direkten chirurgischen Exzision über andere Techniken wie Liposuction bis hin zu Kombinationsverfahren. Hautexzision erfolgt jeweils in Abhängigkeit vom Grad der Gynäkomastie. In der vorliegenden Studie werden die Ergebnisse einer zur chirurgischen Exzision adjuvanten Liposuction beschrieben.

**Patienten und Methoden:** Im Zeitraum September 2012 – April 2015 wurden insgesamt 14 Patienten durch kombinierte Liposuction und Exzision über einen periareolären Zugang behandelt.

In allen Fällen erfolgte eine präoperative Evaluation zum Ausschluss anderweitiger Gynäkomastieursachen.

**Ergebnisse:** Bei allen 14 Patienten erfolgte die Behandlung beidseitig (28 Gewebeexzidate). Das Alter der Patienten lag zwischen 13 und 33 Jahren. Zwei Patienten wurden als Grad I klassifiziert, je vier als Grad IIa, IIb bzw. III. Die ersten drei Patienten entwickelten ein Serom, zwei eine partielle oberflächliche Epidermolyse der Areola. Eine oberflächliche Wundinfektion kam unter konservativer Behandlung zur Ausheilung.

**Schlussfolgerung:** Alle Schweregrade der Gynäkomastie ließen sich mit dem beschriebenen Verfahren behandeln. Bei einem Patienten mit erheblichem Hautüberschuss und schlechtem Hautstatus wurde eine zusätzliche Hautresektion erforderlich.

## Introduction

Gynecomastia is a deformity of male chest due to an enlargement of the male breast. It ranges between 32–36% in living and 40–55% in autopsy of all men [1], [2], [3]. This enlargement can be either due to proliferation of ductal tissue or deposition of fatty tissue or more commonly a combination of both. Gynecomastia can present asymmetrically and often bilaterally. Patients usually have cosmetic and psychological problems [4].

Treatment of gynecomastia varied from direct surgical excision to other techniques. Surgical correction still keeps the main treatment for gynecomastia. Other techniques such as traditional liposuction, ultrasound-assisted liposuction, arthroscopic shaver, mammotome mastectomy or endoscopic procedures were introduced to decrease the morbidity of conventional surgical excision. Unfortunately, neither surgical nor other methods can work alone for correction of all types of gynecomastia [5], [6]. 

In this study, personal experience of using a combination of liposuction and a subsequent surgical excision through a single periareolar incision in a single stage is described.

## Patients and methods

Between September 2012 and April 2015, a total of 14 patients were treated with liposuction and surgical excision through a periareolar incision. 

Preoperative evaluation was done in all cases, to exclude any underlying cause of gynecomastia. If any abnormalities were seen during examination, additional workup was done for the patient radiologically and pathologically.

 Informed consent was obtained from all patients involved in the study. Separate consent for photography was taken from all cases involved in the study.

### Surgical technique

Under general anesthesia, both breasts were infiltrated by tumescent technique (adrenaline concentration 1/500,000). A single superficial periareolar incision was made. An area of 1 cm was deepithelialized to the dermal level. Liposuction was done at a superficial level by suction assisted cannula (no. 4) to separate the skin of the chest from the underlying breast tissue. Then the cannula was used to destruct the inframammary fold.

The cannula was removed and the periareolar incision was deepened. The one cm of deepithelialized tissue together with breast disc (thickness = 1 cm) underlying the areola was preserved. This would prevent depression or saucer deformity of nipple areola complex.

The breast tissue was removed from the underlying muscle without injury of the pectoralis fascia. This was mandatory to prevent future seroma and hematoma formation. Breast tissues could be removed as one peace or into two halves in large breasts. All breast tissues were sent for pathological examination.

Haemostasis was achieved under direct vision. This was ensured by irrigation of the bed with saline. This irrigation revealed bleeding points that were coagulated by electrocautery. Suction drain was inserted through a separate stab in the axilla per breast. Skin sutures and pressure garments were applied.

### Postoperative care

The patient was discharged on 1^st^ day after surgery. Drains stayed till the 3^rd^ postoperative day. Pressure garments were applied on the operating table and were prescribed to be applied for 4 weeks to prevent seroma and hematoma formation.

### Follow-up

All patients were seen in the out-patient clinic every week for one month. Pressure garments were changed every week for 4 weeks. Follow-up of patients was extended up to 2 years.

## Results

**Figure 1:**
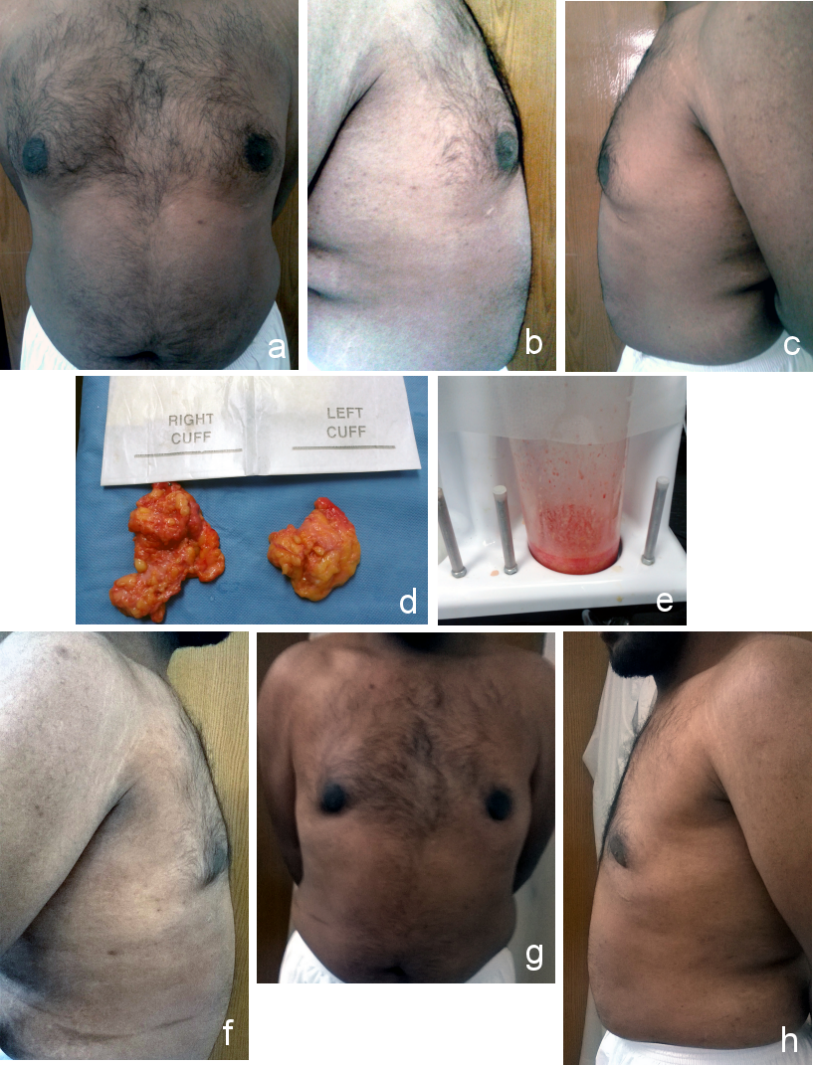
Grade I gynecomastia a) Anterior view (preoperative); b) right lateral view(preoperative); c) left lateral view (preoperative); d) liposuction; e) excised breast tissue; f) anterior view (postoperative after 6 months); g) right lateral view (postoperative after 6 months); h) left lateral view (postoperative after 6 months)

**Figure 2:**
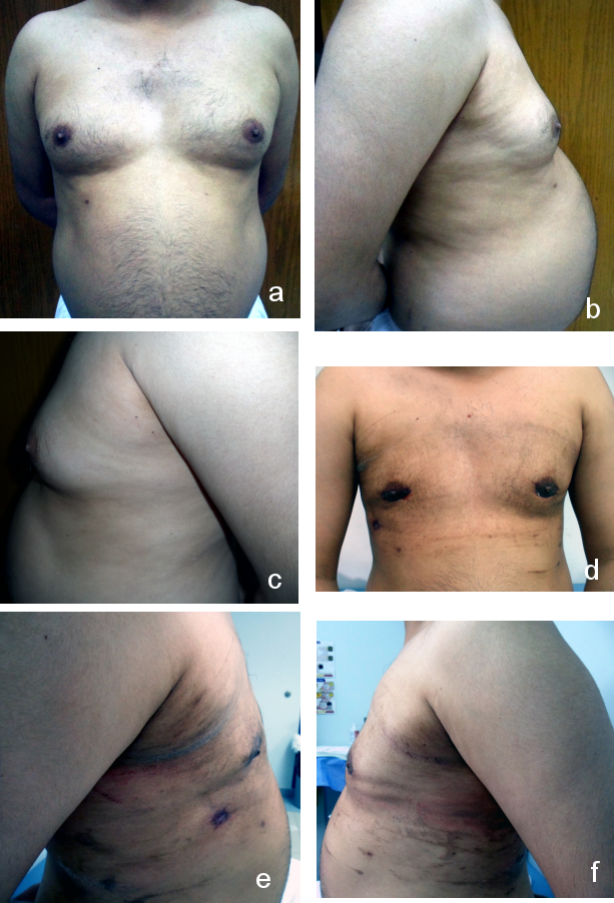
Grade IIa gynecomastia a) anterior view (preoperative); b) right lateral view (preoperative); c) left lateral view (preoperative); d) anterior view (postoperative after 2 months); e) right lateral view (postoperative after 2 months); f) left lateral view (postoperative after 2 months)

**Figure 3:**
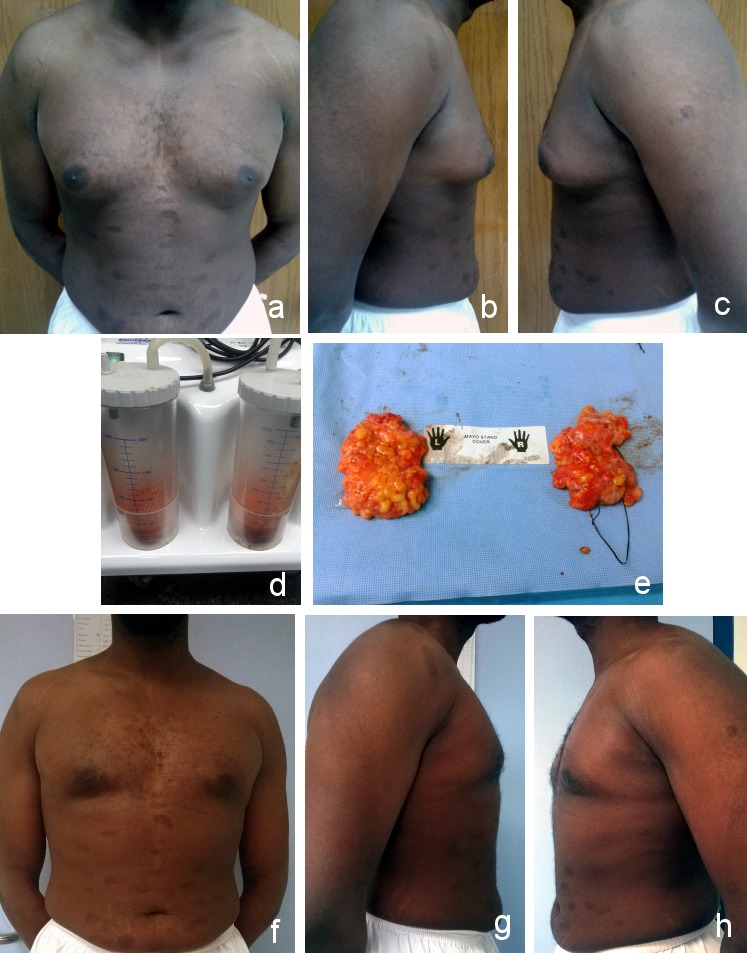
Grade IIa gynecomastia a) anterior view (preoperative); b) right lateral view (preoperative); c) left lateral view (preoperative); d) liposuction; e) excised breast tissue; f) anterior view (postoperative after 9 months); g) right lateral view (postoperative after 9 months); h) left lateral view (postoperative after 9 months)

**Figure 4:**
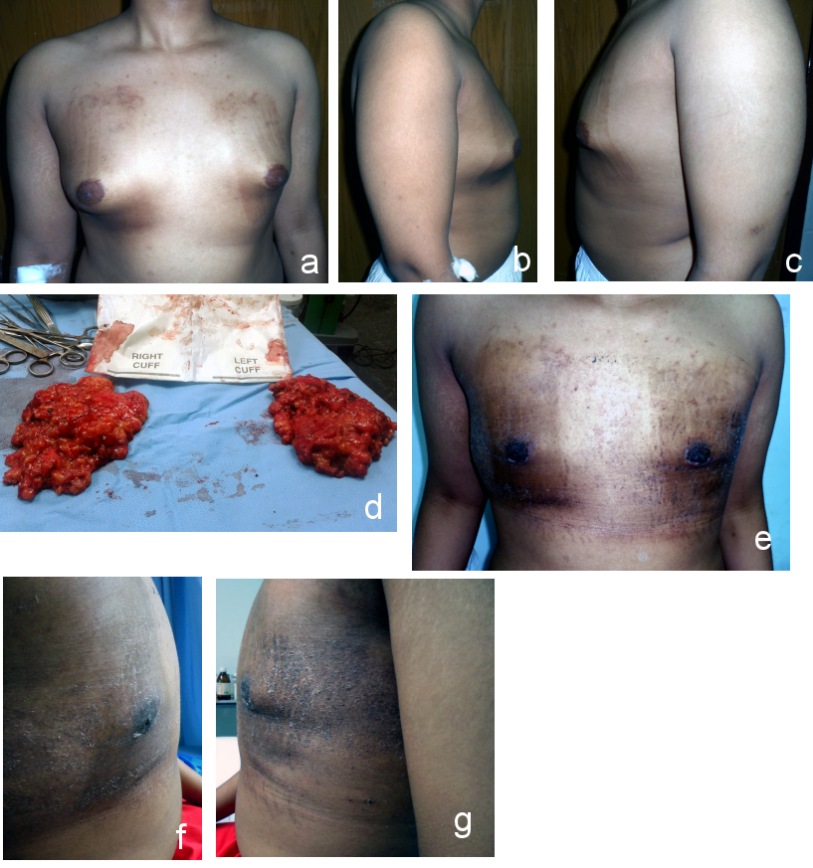
Grade IIb gynecomastia a) anterior view (preoperative); b) right lateral view (preoperative); c) left lateral view (preoperative); d) excised breast tissue; e) anterior view (postoperative after 1 month); f) right lateral view (postoperative after 1 month); g) left lateral view (postoperative after 1 month)

**Figure 5:**
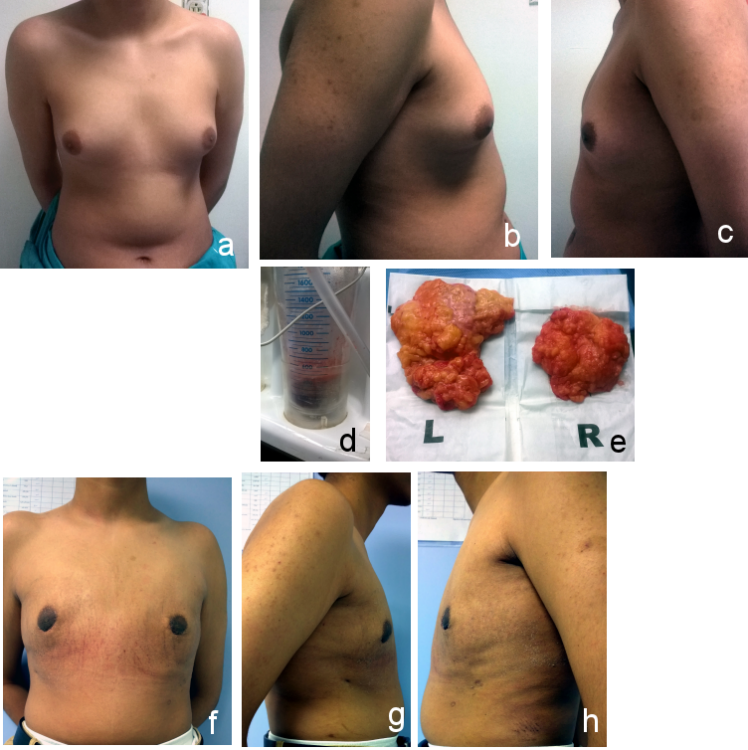
Grade IIb gynecomastia a) anterior view (preoperative); b) right lateral view (preoperative); c) left lateral view (preoperative); d) liposuction; e) excised breast tissue; f) anterior view (postoperative after 11 months); g) right lateral view (postoperative after 11 months); h) left lateral view (postoperative after 11 months)

**Figure 6:**
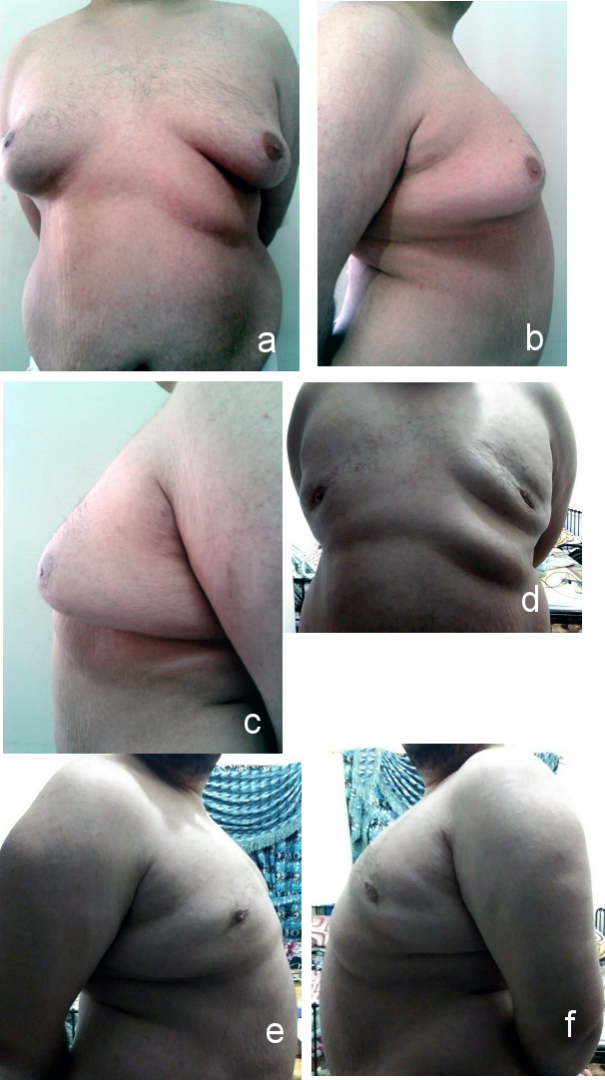
Grade III gynecomastia a) anterior view (preoperative); b) right lateral view (preoperative); c) left lateral view (preoperative); d) anterior view (postoperative after 2 years); e) right lateral view (postoperative after 2 years); f) left lateral view (postoperative after 2 years)

All eighteen patients were treated bilaterally (28 breast tissues). Their ages ranged between 13 and 33 years. The patients were classified as grade I (n=2) (Figure 1 (Fig. 1)), grade IIa (n=4) (Figure 2 (Fig. 2), Figure 3 (Fig. 3)), grade IIb (n=4) (Figure 4 (Fig. 4), Figure 5 (Fig. 5)) and grade III (n=4) (Figure 6 (Fig. 6)). Only one patient aged 33 years was treated with skin excision. Operative time ranged between 35–60 minutes per each breast. Time varied according to the grade of gynecomastia. All cases were one-day case surgery. Pathological examination did not reveal any abnormality. There were no cases presented with nipple-areola complex necrosis. All the patients were satisfied with the results and none of them asked for reoperation.

### Complications

The first 3 patients showed seroma. These cases were treated conservatively by aspiration once a week for 3 weeks boosted by the application of pressure garments. Preservation of pectoralis fascia prevented seroma formation in the following cases. Slight nipple retraction occurred in 3 patients. Partial superficial epidermolysis of areola occurred in 2 cases. Superficial infection occurred in one case and was treated conservatively.

### Case with skin excision

**Figure 7:**
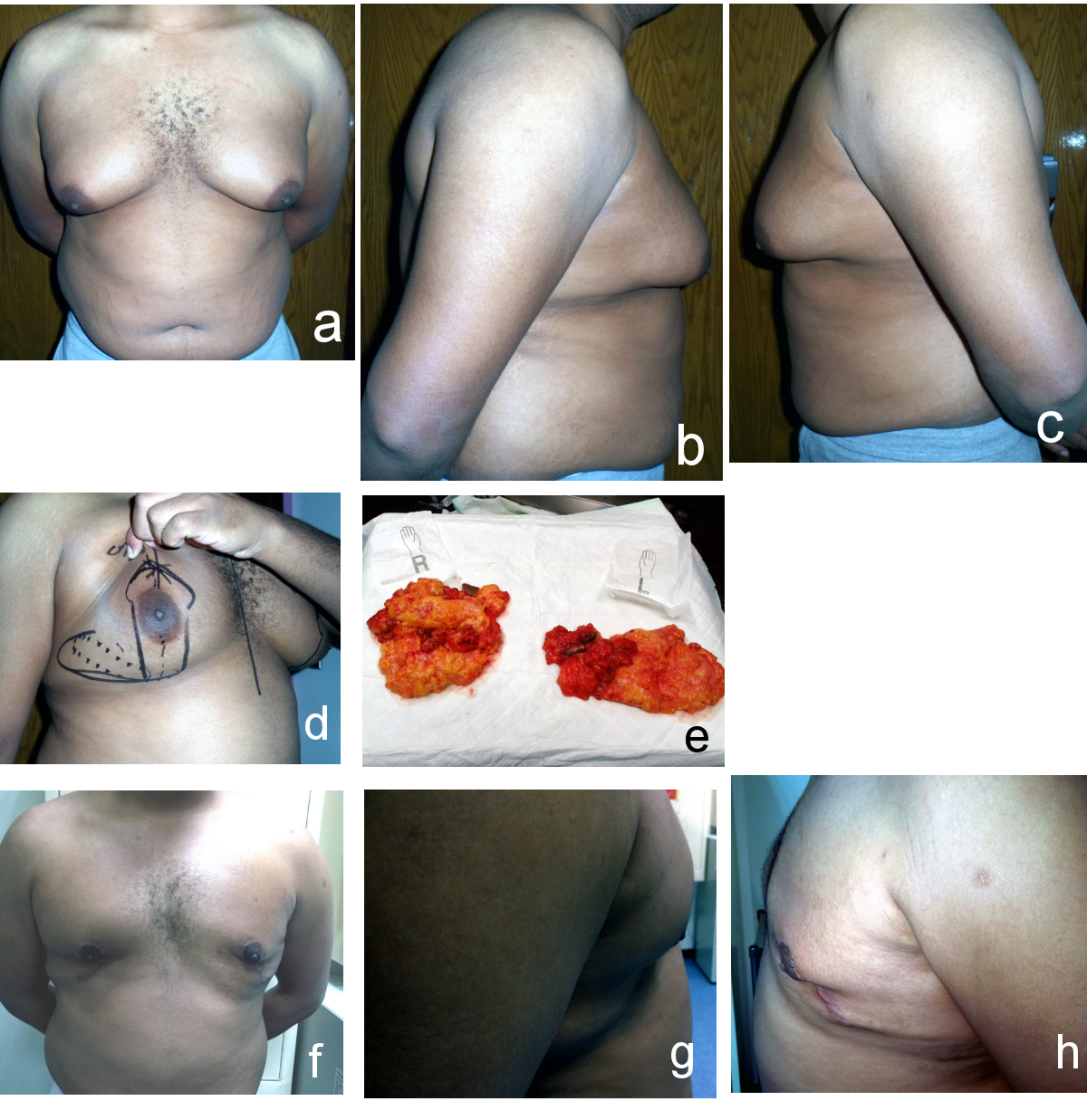
Grade III gynecomastia with skin excision a) anterior view (preoperative); b) right lateral view (preoperative); c) left lateral view (preoperative); d) marking of incision; e) excised breast tissue; f) anterior view (postoperative after 9 months); g) right lateral view (postoperative after 9 months); h) left lateral view (postoperative after 9 months)

A 33-year-old male presented with a grade III gynecomastia. The patient had a history of stopping swimming activity 3 years ago. He built-up about 15 kg in the last year. By examination, the patient had severe redundancy and bad skin complexion. A workup ruled out any pathological cause. Skin excision was decided (Figure 7 (Fig. 7)). The incision was discussed with the patient. The patient chose the vertical incision rather than other incisions. Liposuction and breast tissues excision were performed. The nipples were elevated to 22 cm from the mid clavicular line. The healing was uneventful. The patient was satisfied with his new appearance. He was followed up for 9 months.

## Discussion

Most incisions for approaching gynecomastia are designed on/and around the areola. In 1946, intraareolar incision was executed by Webster [7]. Transverse areolar incision was first described by Pitanguy for gynecomastia [8]. The incision was then modified from passing through the nipple to reverse omega that can be extended to H shaped incision [9]. Actually, any incision through the areola was not accepted by our patients. In addition, hypopigmentation and limited exposure might occur.

The periareolar incision was described originally by Dufourmentel in 1928 [10]. This incision affords a better access to all quadrants of the breast tissue and gives a good aesthetic appearance. In this work, a periareolar incision was used to perform liposuction. From the same incision, surgical excision was performed. Indeed, the periareolar scar was considered small and inconspicuous. It allowed access to all quadrants of the breast and subsequently adequate excision and better hemostasis.

Needless to say, the aim of any classification is to guide for the way of adequate management. Simon’s classification [11] is the most adopted classification of gynecomastia. This classification was used in this study. 

In 2005, Hammond and his colleagues described a combined technique using ultrasound assisted liposuction combined with surgical excision [12]. This was followed in 2007 by a group from the Netherlands, where they used suction assisted liposuction combined with surgical excision. This was augmented by ultrasonic assisted liposuction [13]. The principle was the same in both articles. Removal of breast tissue was carried out mainly by liposuction and any residual tissue was removed by surgical excision.

**Figure 8:**
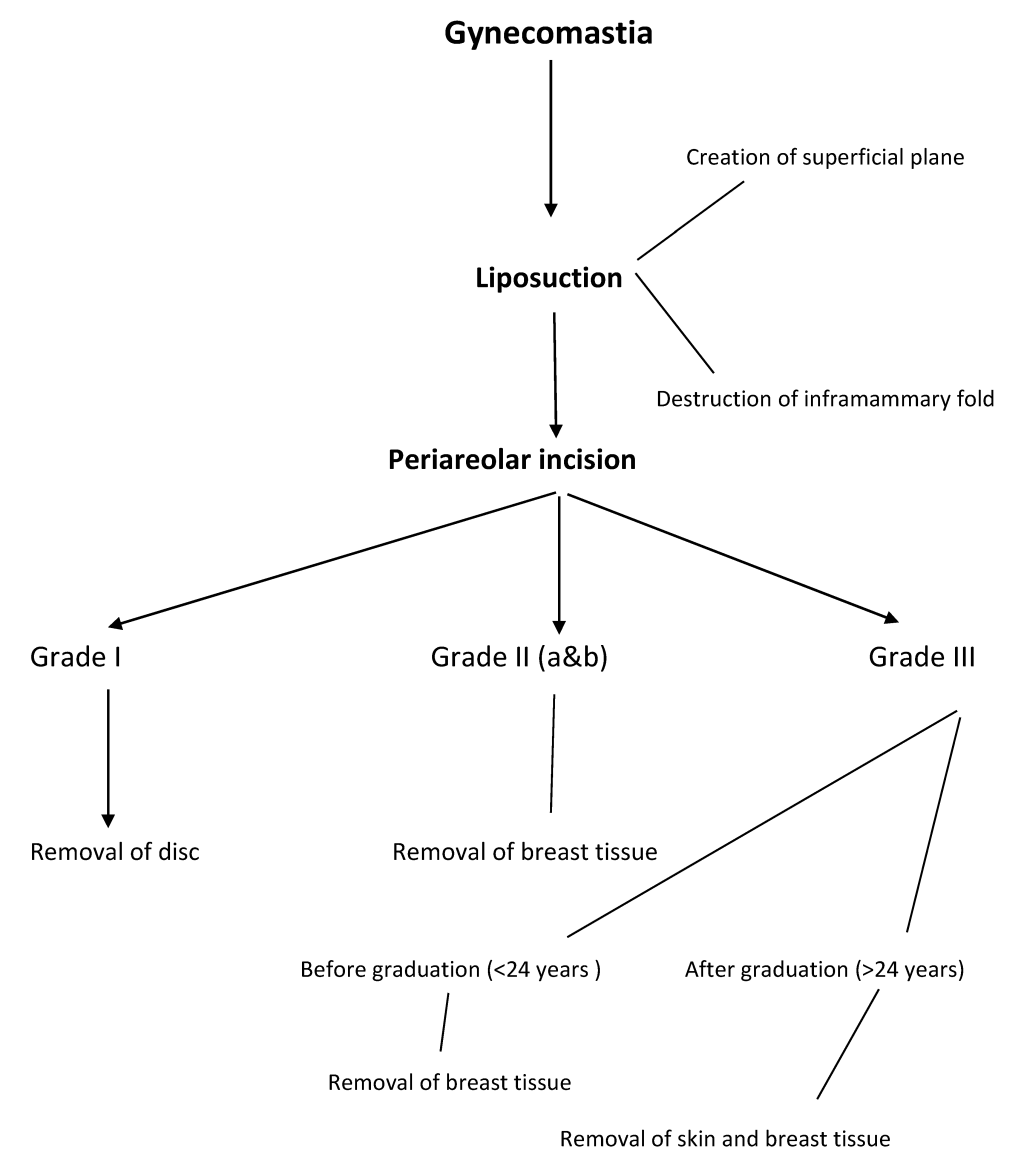
Algorithm for management of gynecomastia

In this article, surgical excision was the main line of treatment and was used in all grades of gynecomastia (Figure 8 (Fig. 8)). Even in grade I, liposuction alone could not remove dense retroareolar tissue. The results were confirmed by a work from Germany [14]. However, in the present work: 1) all grades of gynecomastia were included and 2) a single surgeon experience was represented. 

Suction-assisted liposuction was used as a mean for simplifying surgical excision, but not for treatment of gynecomastia. Usage of liposuction prevented inadvertent injury of skin during dissection and created a superficial plane between skin and breast tissue. This superficial plane facilitated excision of breast tissue. Also, liposuction was used for destruction of inframammary fold and merging of chest skin with abdominal skin. This merging eliminated the need for skin excision in grades IIB and III. Moreover, skin contractility was enhanced by liposuction.

The skin of younger patients has an excellent ability to contract after removal of breast tissue [6]. Fortunately, most patients were presented in adolescent period. Skin excision was done only to a patient who presented at 33 years of age. Change in his lifestyle and stoppage of outside activities were contributing factors for his skin excess. Vertical skin excision was used [6]. It was preferred to inverted T incision or inframammary incision. Good exposure, adequate excision, acceptable scar, and ability for repositioning of nipple were the main merits.

Drains were routinely used in this work. Patients were allowed to discharge on the 1^st^ day following the operation to appear in the clinic after three days. In all patients, there was no need to keep the patient hospitalized till removal of drains.

The most serious complication was seroma. It occurred in the first three cases. Aspiration once a week for 2–3 weeks resolved the problem together with the help of pressure garments. 

One limitation of this technique was the limited number of patients. However, as all patients were operated by a single surgeon, this disadvantage can be forgiven.

In summary, surgical excision combined with liposuction through a single periareolar incision in a single stage was a safe and a fruitful technique for the management of any grade of gynecomastia with good aesthetic results and a low complication rate.

## Conclusion

Simplicity and productivity are signs of any successful technique. All grades of gynecomastia were corrected by the same approach. Skin excision was added to a patient that had sever skin excess with limited activity and bad skin complexion. No patients required another setting or asked for 2^nd^ opinion. Combined approach was a versatile and less complicated method for treatment of gynecomastia.

## Notes

### Level of evidence

IV: Cases series.

### Ethical standards 

This study has been performed in accordance with the ethical standards set forth in the 1964 Declaration of Helsinki and its later amendments. Informed consent was obtained from all parents responsible for participants included in the study.

### Competing interests

The author declares that he has no competing interests.
